# Efficacy of androgen deprivation therapy (ADT) in combination with radiation therapy, compared to ADT alone in patients with high-risk prostate cancer: an updated systematic review and meta-analysis

**DOI:** 10.25122/jml-2025-0140

**Published:** 2026-02

**Authors:** Walaa Borhan, Ahmad Assinnari, Abdulaziz Bakhsh, Mansour Alnazari, Emad Rajih

**Affiliations:** 1 Department of Basic Medical Sciences, College of Medicine, Taibah University, Madinah, Saudi Arabia; 2 Department of General and Specialized Surgery, College of Medicine, Taibah University, Madinah, Saudi Arabia

**Keywords:** prostate cancer, node positive, locally advanced, androgen deprivation, radiotherapy, survival, progression, complications, meta-analysis, systematic review

## Abstract

Androgen deprivation therapy (ADT) has long been a cornerstone of treatment for patients with locally advanced or metastatic hormone-sensitive prostate cancer. The efficacy of ADT plus radiotherapy (RT) compared to ADT alone remains unclear due to conflicting results in existing literature. The study aimed to systematically evaluate the effectiveness of ADT combined with RT versus ADT alone in patients with prostate cancer (clinically node positive, locally advanced disease, metastatic disease), focusing on overall survival (OS), prostate-specific mortality (PSM), progression-free survival (PFS), and the risk of complications. A comprehensive search of PubMed, Embase, Web of Science, and Scopus was conducted between 1^st^ January 2000 and 15^th^ October 2024 to identify studies comparing ADT alone to ADT combined with RT. Hazard ratios (HRs) and relative risks (RRs) with 95% confidence intervals (CIs) were calculated for the outcomes. The certainty of the evidence was assessed using the standard GRADE approach. A total of 8 studies met the inclusion criteria (6 RCTs and 2 cohort studies). These studies included 18,456 patients. The combination of ADT and RT significantly improved OS (HR = 0.75, 95% CI, 0.63, 0.90), PFS (HR = 0.41, 95% CI, 0.20, 0.84), and reduced PSM (HR = 0.52, 95% CI, 0.34, 0.78) compared to ADT alone. Subgroup analysis showed greater OS (HR = 0.66, 95% CI, 0.59, 0.75) and PSM (HR = 0.43, 95% CI, 0.39, 0.49) in patients with locally advanced or node-positive disease. ADT + RT was also associated with increased risks of genitourinary (RR = 1.80, 95% CI, 1.15, 2.82), gastrointestinal (RR = 4.18, 95% CI, 1.46, 11.96), and sexual dysfunction-related complications (RR = 1.10, 95% CI, 1.02, 1.18). The overall certainty of evidence was judged to be 'moderate' for survival outcomes and 'low' for risk of complications. Combining ADT with radiation therapy RT significantly improved survival, compared to ADT alone, especially in patients with locally advanced or node-positive prostate cancer, yet with moderate GRADE certainty. However, this combination also increased the risk of complications. The results advocate that our findings are most applicable to high-risk non-metastatic and cN+ disease and do not support routine addition of RT to ADT in unselected metastatic patients. Therefore, further research is needed to refine treatment protocols and identify the optimal timing and patient subgroups for this approach.

## INTRODUCTION

Prostate cancer is one of the most prevalent cancers among men globally [1]. In 2020, there were an estimated 1.41 million new cases and 375,000 deaths from prostate cancer worldwide [2]. From 1990 to 2019, the incidence and mortality rates of prostate cancer increased by nearly 116.0% and 109.0%, respectively, a trend likely to continue as the global population ages [2]. Over the past few decades, treatment strategies for prostate cancer have evolved significantly. For patients with locally advanced or metastatic hormone-sensitive prostate cancer, androgen deprivation therapy (ADT) has remained a cornerstone of treatment [3,4]. By inhibiting androgen production, ADT helps slow disease progression and improves control [5]. However, despite its effectiveness, ADT alone often fails to provide long-term remission, particularly in patients with more advanced stages of the disease [6].

Recent studies have investigated combining ADT with other treatment options, such as radiation therapy (RT), to improve survival outcomes [7-9]. Radiation therapy targets localized tumour areas, aiming to reduce cancer progression and enhance overall survival. Randomized controlled trials have compared ADT alone to ADT combined with RT, yielding mixed results. While some studies report a notable survival benefit with the combined treatment [10,11], others either did not show any survival benefit or raised concerns about potential side effects [12,13].

Previous efforts have attempted to synthesize the findings of studies comparing ADT alone to ADT combined with RT, but the evidence has not been conclusive [8,14,15]. Wang *et al*. conducted a meta-analysis of two randomized controlled trials comparing a combination of ADT and RT with ADT monotherapy, finding no significant difference in overall or progression-free survival, except for improved survival in patients with low-volume disease [14]. Similarly, Lei *et al*.'s review of three RCTs found that adding RT to long-term ADT improved overall survival in patients with locally advanced prostate cancer [15]. However, these reviews did not perform appropriate subgroup analyses to determine whether the effects on survival and disease progression differ across patient and disease characteristics. Since the publication of these reviews, more studies have been conducted on this topic, necessitating an update of the existing evidence. Thus, the current meta-analysis aimed to comprehensively evaluate and compare the effectiveness of ADT combined with RT versus ADT alone, focusing on primary outcomes including overall survival, prostate-specific mortality, and progression-free survival. We further aimed to include more recent studies published up to 15 October 2024 that were not available in earlier meta-analyses by Wang *et al*. [14] and Lei *et al*. [15]. In addition, more granular subgroup analyses based on disease extent (locally advanced prostate cancer [LAPC] or clinically node-positive disease [cN] versus metastatic disease), baseline prostate-specific antigen (PSA) levels, and Gleason score were planned to better identify patient subgroups that derive the greatest survival benefit from combined ADT and RT.

## MATERIAL AND METHODS

### Compliance with relevant guidelines

Our meta-analysis was conducted in accordance with the Preferred Reporting Items for Systematic Reviews and Meta-Analyses (PRISMA) guidelines [16]. In addition, the study protocol was prospectively registered in PROSPERO (https://www.crd.york.ac.uk/prospero/) under registration number CRD42024602727.

### Identification of studies

A systematic and thorough search strategy was developed to identify relevant studies from electronic databases, including PubMed, Embase, Web of Science, and Scopus. The specific search strategy for each of the above databases is presented in Supplementary Table 1. The search was limited to studies published between 1^st^ January 2000 and 15^th^ October 2024. Manual searches of reference lists and relevant review articles were conducted to ensure the inclusion of any additional studies that may have been overlooked during the electronic search.

Supplementary File

**Table 1:** Summary characteristics of the studies included in the review

Author (publication year)	Study design and place of study	Age	Tumour characteristics	Intervention(I) and comparator (C)	Sample size	Duration of follow up
Lee TH *et al*. (2024) [22]	RCT; Republic of Korea	Median age similar in both groups (70 years)	T3/T4 (100%); cN+; median PSA (36 ng/ml); node positive but no distant metastasis Gleason score of 8-10 (85%)	C: ADT administered using a combination of a gonadotropin-releasing hormone (GnRH) agonist and anti-androgen. ADT given for at least 2 years I: ADT along with radiotherapy (RT) (70 Gy in 28 fractions). RT started after 2-3 months of ADT	60 I: 29 C: 31	Median 3.3 years
Sargos *et al*. (2020) [13]	RCT; France	Mean age similar in both group (~71 years)	LAPC; T3N0M0 (95%); median PSA of 26.8 ng/ml; PSA (≥20 ng/ml) in ~64% Gleason score of 7 or less (83%)	C: LHRH agonist (leuprorelin) for 3 years, along with oral flutamide (750 mg/day) for the first month. I: In addition to ADT, external beam radiation therapy (EBRT) (whole pelvis dose of 46 Gy with prostate having boost from 20 to 28 Gy), initiated within 90 days of the first leuprorelin injection	263 I: 133 C: 130	Median 7.3 years
Boevé *et al*. (2019) [23]	RCT; Netherlands	Mean age similar in both group (~67 years)	T3 (82%) with osseous metastasis; median PSA of around 135 ng/ml; PSA (>20 ng/ml) in all; Gleason score of 7 or more (>90%)	C: Bicalutamide (50 mg daily) for 4 weeks as flare reduction, followed by LHRH agonist starting 1–2 weeks after randomization, continued until death. I: In addition to ADT, EBRT started within 3 months of ADT initiation, with a prescribed dose of 70 Gy	432 I: 216 C: 216	Median 47 months
Parker *et al*. (2018) [12]	RCT; Multicentric (Switzerland and UK)	Median age similar in both group (~68 years)	T3/T4 (82%); N+ (64%); median PSA (98 ng/ml); with predominantly high metastatic burden; Gleason score of 8-10 (82%)	C: lifelong androgen deprivation therapy as either gonadotrophin-releasing hormone agonists or antagonists or orchidectomy. I: ADT along with external-beam radiotherapy in one of the two ways: either 36 Gy in six consecutive weekly fractions of 6 Gy, or 55 Gy in 20 daily fractions of 2.75 Gy over 4 weeks. Median time of 75 days for start of RT from the time of ADT initiation	2061 I: 1,032 C: 1,029	Median 37 months
Mason *et al*. (2015) [11]	RCT; Multicentric (UK and Canada)	Median age similar in both groups (69.7 years)	LAPC; T3/T4 (87%); PSA (>20 ng/ml) in 63% subjects Gleason score of <8 (63%)	All patients received lifelong ADT before randomisation (patients chose between bilateral orchiectomy (7%) or LHRH agonist (93%)) I: RT was started within 8 weeks of randomisation (65–69 Gy to the prostate and seminal vesicles, 45 Gy to pelvic nodes)	1205 I: 603 C: 602	Median 8.0 years
Lin CC *et al*. (2015) [24]	Retrospective cohort; USA Propensity score matching done	Median age of subjects 66 years in both groups	T1/T2 (58%); clinically node positive, cN+; PSA (>20 ng/ml) in 49% subjects; Gleason score of 8-10 (64%)	C: Data on type and duration of ADT not provided I: ADT along with external beam radiotherapy with median doses of 50.4 Gy to the pelvis and 75.6 Gy total	636 I: 318 C: 318	Median follow-up period varied from 2.7 to 5.2 years
Bekelman *et al*. (2015) [25]	Retrospective cohort; USA Propensity score matching done	Mean age of 71 years in both groups	T2 (90%) WHO grade 2 or Gleason score 5-7 (65%)	C: ADT was defined as orchiectomy or ≥1dose of a gonadotropin-releasing hormone agonist within the first 9 months of diagnosis I: ADT with RT	12,924 I: 8,282 C:4,642	Mean follow-up 6 years
Widmark *et al*. (2009) [10]	RCT; Multicentric (Norway, Sweden, Denmark)	Mean age similar in both groups (~66 years)	LAPC; T3N0M0 (78%); median PSA of 16.0 ng/ml PSA <20 ng/ml (60%) WHO grade 2 (65%)	C: Total androgen blockade with LHRH-agonist for 3 months, along with simultaneous oral flutamide (250 mg, three times daily); Continued flutamide after 3 months until progression or death I: Same total androgen blockade and flutamide treatment for the first 3 months; After 3 months, radiotherapy (≥70 Gy) initiated while continuing flutamide until progression or death.	875 I: 436 C: 439	Median 7.6 years

RCT, randomized controlled trial; LAPC, locally advanced prostate cancer; PSA, prostate-specific antigen; cN+ indicates clinically node-positive prostate cancer

### Inclusion and exclusion criteria

Studies involving adult patients (aged 18–75 years) with prostate cancer, confirmed through pathological evaluation, were eligible for inclusion. Eligible studies required an intervention group receiving a combination of ADT and radiation therapy, compared to a control group receiving ADT alone. Studies were excluded if participants had undergone other baseline treatments, such as radical prostatectomy. The primary outcomes of interest included overall survival (OS), progression-free survival (PFS), and prostate/cancer-specific mortality (PSM). The secondary outcome of interest was the risk of complications. Only studies with a randomized controlled trial (RCT), cohort, or case-control design were considered, and studies needed to report effect sizes with 95% confidence intervals for at least one outcome to ensure data relevance. Inclusion was restricted to English-language studies published in peer-reviewed journals. When multiple studies originated from the same dataset, the study with the longest follow-up period was selected for inclusion.

Studies involving patients younger than 18 or older than 75 years were excluded. Additionally, studies were not eligible if the comparison groups were irrelevant to the review objective, such as comparisons of ADT with radiation therapy versus RT alone, studies assessing ADT as concurrent versus neoadjuvant therapy with RT, or comparisons based on varying durations of ADT or different durations and doses of RT. Case reports, case series, review articles, editorials, letters to the editor, and commentaries were also excluded. Studies that did not report usable effect sizes for analysis were not considered.

### Process of study selection

After applying the search strategy across designated databases, the first step was to remove duplicate studies. Two independent experts (ASR, WMB) then conducted a thorough review of the remaining studies. In the initial screening phase, each study’s title and abstract were carefully examined to assess their relevance to the research question. Studies that appeared potentially relevant were selected for further evaluation. In the next stage, a detailed assessment of the full text was performed to determine each study’s eligibility for inclusion in the meta-analysis. Any disagreements regarding study inclusion were resolved through in-depth discussions among the authors. During the initial screening phase, an inter-rater reliability assessment was also conducted among the reviewers (ASR, WMB) to evaluate consistency in study selection. If the initial agreement was found to be below the acceptable level (inter-rater reliability below 75%), a detailed discussion was held with the screening team, led by the principal investigator (ESR), to ensure clarity and alignment on inclusion and exclusion criteria.

### Data extraction and quality assessment

Data extraction from the final set of included studies was conducted independently by two authors using a standardized data extraction form. Extracted data included study identifiers (first author’s name and year of publication), study design, country of study, participant and tumour characteristics, management details for the intervention and control groups, sample size, follow-up duration, and relevant findings. Any discrepancies between the two authors were resolved through discussion to reach a consensus. For risk of bias assessment, we used the Newcastle-Ottawa Scale (NOS) for cohort studies and the Cochrane Risk of Bias (RoB 2.0) tool for RCTs [17,18]. Quality assessment was performed independently by the two independent experts (MN, WB). The certainty of the evidence was assessed using the GRADE approach (gradepro.org) and GRADEpro software [19].

### Statistical analysis

Statistical analysis was performed by RevMan (Version 7.2.0. The Cochrane Collaboration, 2024, available at *revman*.cochrane.org). Survival outcomes (OS, PFS, PSM) were pooled using hazard ratios reported or derived from both RCTs and cohort studies, each with corresponding 95% confidence intervals (CI). Complication outcomes were analysed separately using relative risks. A random-effects model was used for all analyses to account for variations in baseline characteristics across studies [20]. The pooled analysis primarily included RCTs, with two cohort studies that used propensity score matching for baseline comparability. A sensitivity analysis was conducted, excluding these cohort studies. Publication bias was assessed using Egger's test and by visually inspecting funnel plots [21]. Statistical significance was defined as a *P* value < 0.05. Subgroup analysis for the primary outcomes was conducted based on the type of prostate cancer (locally advanced prostate cancer, LAPC or node positivity, cN+ and metastatic), baseline Gleason score (≤7 and ≥8), baseline PSA level (≥20 ng/ml or <20 ng/ml), and sample size (<500 and ≥500).

Meta-regression was not prespecified in the PROSPERO protocol and, given the inclusion of only eight studies, would likely be underpowered and potentially unstable. To avoid post-hoc, data-driven inference, we did not perform meta-regression. Instead, heterogeneity was explored using prespecified subgroup and sensitivity analyses (by stage, Gleason score, baseline PSA, and sample size), which are now more clearly presented and discussed as exploratory tools.

## RESULTS

**Figure 1:**
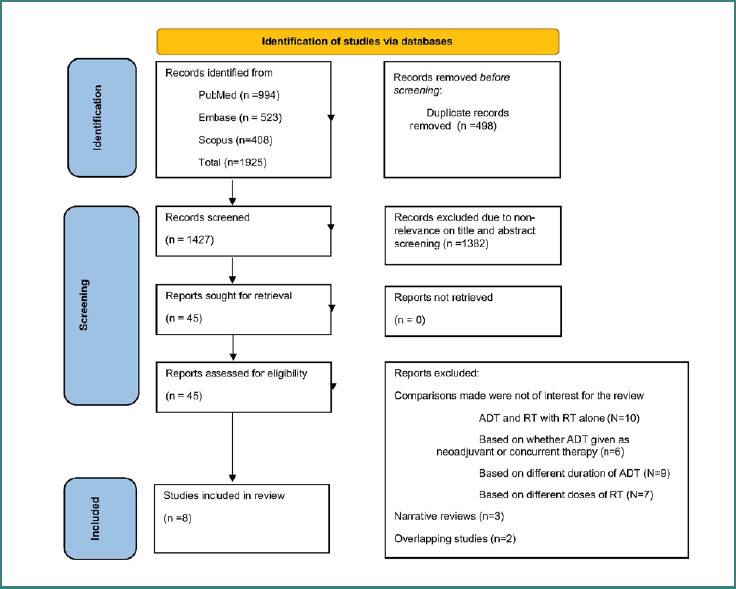
Selection process of studies included in the review

We conducted a systematic search and identified 1,925 studies. After removing 498 duplicates, we screened the titles and abstracts of the remaining 1,427 unique studies. This led to the exclusion of 1,382 studies based on their potential relevance to the review. We then reviewed the full texts of the remaining 45 studies and excluded 37 more that did not meet our inclusion criteria, as shown in Figure 1. This process resulted in a final selection of eight studies for analysis [10-13, 22-25].

The study characteristics are provided in Table 1. There were six RCTs [10-13, 22,23]. In the remaining two cohort-based studies, propensity score matching was used, ensuring that baseline characteristics between the study groups were similar [24,25]. Three studies were multicentric; two were conducted in the United States of America (USA), and one each was conducted in the Republic of Korea, France, and the Netherlands (Table 1). The two cohort studies obtained a score of 7 (out of the maximum attainable score of 9) on the NOS assessment (Supplementary Table 2). The remaining five RCTs were also judged to have low risk of bias (Supplementary Figure 1).

**Table 2:** Findings from the subgroup analysis comparing ADT and RT, with ADT alone

	Overall survival (OS)	Prostate-specific mortality (PSM)	Progression-free survival (PFS)
	HR (95% CI); (*n*, I^2^)		
**Type** LAPC or cN+ Metastatic	0.66 (0.59, 0.75) (5, 51.6%) 0.98 (0.82, 1.17) (2, 39.8%)	0.43 (0.39, 0.49) (4, 0.0%) 0.93 (0.80, 1.09) (1, ---)	0.24 (0.15, 0.37) (3, 86.7%) 0.94 (0.84, 1.04) (2, 0.0%)
**Gleason score at baseline** ≤7 ≥8	0.68 (0.61, 0.77) (4, 44.4%) 0.81 (0.55, 1.19) (3, 88.7%)	0.43 (0.39, 0.49) (4, 0.0%) 0.93 (0.80, 1.09) (1, ---)	0.24 (0.15, 0.37) (3, 86.7%) 0.94 (0.84, 1.04) (2, 0.0%)
**PSA at baseline** High (≥20 ng/ml) Low (<20 ng/ml)	0.88 (0.75, 1.04) (4, 60.6%) 0.59 (0.43, 0.79) (2, 55.9%)	0.58 (0.32, 1.05) (3, 90.5%) 0.44 (0.30, 0.65) (1, ---)	0.52 (0.28, 0.97) (4, 97.0%) 0.16 (0.12, 0.21) (1, ---)
**Sample size** <500 ≥500	0.97 (0.72, 1.30) (2, 57.0%) 0.69 (0.57, 0.84) (5, 85.7%)	0.40 (0.20, 0.80) (1, ---) 0.54 (0.34, 0.85) (4, 94.8%)	0.49 (0.16, 1.52) (2, 95.7%) 0.36 (0.12, 0.97) (3, 99.0%)

HR, hazard ratio; LAPC, locally advanced prostate cancer; cN+, clinically node positive prostate cancer

The average age of the subjects in both groups (i.e., ADT+RT and ADT alone) was similar across all included studies and ranged from 66 to 71 years. There were four studies with participants having LAPC [10,11,13,25], two studies with clinically node positive prostate cancer (cN+) [22,24], and the remaining two studies had subjects with distant metastasis [12,23]. The included studies contributed to a sample of 18,456 subjects, with 11,049 receiving ADT with RT and 7,407 receiving only ADT. All studies had long-term follow-up (ranging from 2.7 to 8.0 years). In all studies except two, RT was initiated within 2-3 months of starting ADT (Table 1). In the two remaining studies, Lin *et al*. [24] and Bekelman *et al*. [25], information regarding the timing of RT initiation relative to the start of ADT was not reported. The definitions of OS and PSM were largely similar across the included studies; however, the definition of PFS varied. Three studies, i.e., Mason *et al*. [11], Parker *et al*. [12], and Sargos *et al*. [13], defined PFS based on any of these: biochemical progression (rising PSA levels), locoregional progression (expansion to nearby tissues or a larger prostatic area), or metastatic progression. On the other hand, Widmark *et al*. [10] and Boevé *et al*. [23] relied solely on rising PSA levels.

### Overall survival (OS), prostate-specific mortality (PSM), and progression-free survival (PFS)

**Figure 2:**
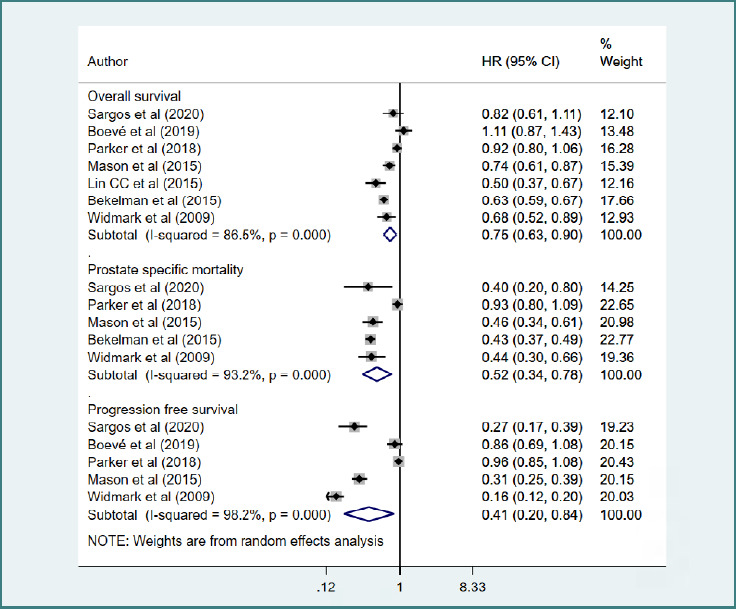
Overall survival (OS), progression free survival (PFS) and prostate specific mortality (PSM) in those receiving a combination of ADT and RT, compared to those with ADT alone

Essentially, PFS definitions varied substantially across studies: three trials (Mason *et al*. [11], Parker *et al*. [12], and Sargos *et al*. [13]) used a composite of biochemical, locoregional, and metastatic progression, whereas two trials (Widmark *et al*. and Boev *et al*.) relied on PSA-based biochemical progression only. Compared to those who received only ADT, those receiving a combination of ADT and RT had improved OS (HR = 0.75, 95% CI, 0.63, 0.90; *n* = 7, I^2^ = 86.5%) and PFS (HR = 0.41, 95% CI, 0.20, 0.84; *n* = 5, I^2^ = 98.2%) (Figure 2). The addition of RT to ADT also significantly reduced the risk of PSM (HR = 0.52, 95% CI, 0.34, 0.78; *n* = 5; I^2^ = 93.2%) compared with ADT alone (Figure 2). There was no evidence of publication bias, both on Egger’s test (*P* = 0.29 for OS; *P* = 0.70 for PSM; *P* = 0.15 for PFS) and on visual inspection of the funnel plots (Supplementary Figures 2-4).

**Figure 3:**
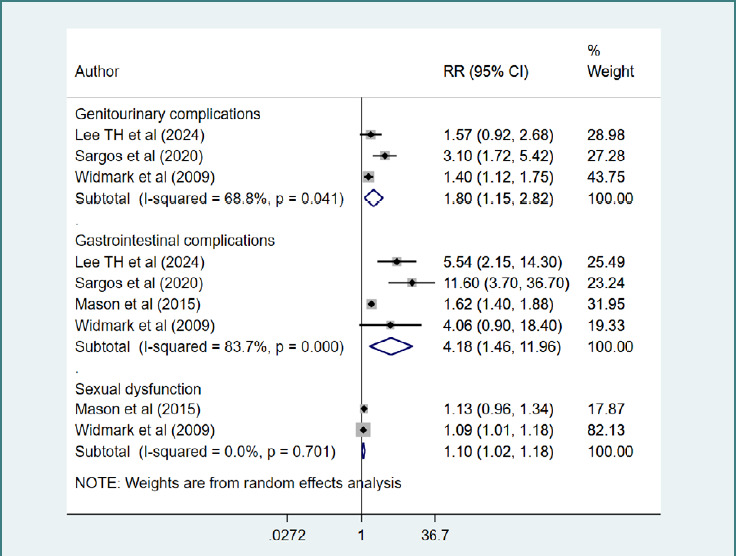
Risk of complications in those receiving a combination of ADT and RT, compared to those with ADT alone

The overall certainty of evidence for these outcomes was judged to be 'moderate' according to the GRADE assessment criteria (Table 3 in the supplementary materials). This is primarily because of:

1. The evidence base was dominated by multiple randomized trials with generally low risk of bias.
2. The direction of effect was consistent across studies, subgroups, and RCT-only sensitivity analyses, and pooled confidence intervals for OS and PSM did not cross unity. Sensitivity analyses were performed, yet restricted to RCTs only for OS and PSM. After excluding the two propensity-matched cohort studies, the survival benefit of ADT+RT remained statistically significant (HR = 0.84, 95% CI, 0.72, 0.99; *n* = 5, I^2^ = 62.6%) and PSM (HR = 0.54, 95% CI, 0.33, 0.89; *n* = 4, I^2^ = 89.4%) (Supplementary Figure 5).

The subgroup analysis shows that patients with LAPC or cN+ disease experienced improved overall survival (HR = 0.66, 95% CI, 0.59, 0.75; *n* = 5, I^2^ = 51.6%) and lower prostate-specific mortality (HR = 0.43, 95% CI, 0.39, 0.49; *n* = 4, I^2^ = 0.0%) with a combination of ADT and RT, but not those with metastatic disease, who had no significant survival benefit (Table 2). Those with lower Gleason scores (≤7) and baseline PSA levels (<20 ng/ml) had better overall survival and progression-free survival outcomes with ADT and RT, suggesting these factors could serve as favourable prognostic indicators (Table 2). Larger studies (≥500 participants) consistently demonstrated survival benefits with a combination of ADT and RT (HR = 0.69, 95% CI = 0.57-0.84; *n* = 5; I^2^ = 85.7%), whereas smaller studies did not show significant survival benefits. Overall, the findings indicate that patients with LAPC/cN+, lower Gleason scores, and lower baseline PSA tend to have better survival and disease progression outcomes when treated with ADT and RT (Table 2).

### Risk of complications

Compared to those who received only ADT, those receiving a combination of ADT and RT had an increased risk of genitourinary (RR = 1.80, 95% CI, 1.15, 2.82; *n* = 3, I^2^ = 68.8%) and gastrointestinal complications (RR = 4.18, 95% CI, 1.46, 11.96; *n* = 4, I^2^ = 83.7%) along with sexual dysfunction (RR = 1.10, 95% CI, 1.02, 1.18; *n* = 2, I^2^ = 0.0%) (Figure 3). There was no evidence of publication bias, both on Egger’s test (*P* = 0.43 for genitourinary complications; *P* = 0.08 for gastrointestinal complications) and on visual inspection of the funnel plots (Supplementary Figures 6-7). The publication bias for sexual dysfunction could not be assessed due to very few studies reporting this outcome. The common complications reported in the included studies were bladder obstruction, urethral stricture, urinary incontinence, increased urinary frequency, moderate to severe diarrhoea, rectal bleeding, proctitis, loss of libido, and erectile dysfunction. The overall certainty of evidence for outcomes related to the risk of complications was judged to be 'moderate' according to the GRADE assessment criteria (Table 3 in the supplementary materials).

## Discussion

The moderate certainty of findings from our meta-analysis is suggestive of the effectiveness of ADT in combination with RT compared to ADT alone for patients with prostate cancer. The survival outcomes are rated as 'moderate certainty,' primarily due to downgrading for serious inconsistency, imprecision, and the limited number of contributing studies. This analysis, encompassing eight studies with around 18,000 participants, revealed significant improvements in OS, PSM, and PFS with the combined treatment approach, particularly in patients with LAPC and cN+ disease. However, the analysis also underscores the increased risk of complications associated with the combination therapy, necessitating careful consideration of treatment strategies in clinical practice. Given the moderate certainty of the evidence, more robust studies are needed to support the findings.

Previous meta-analyses have examined the efficacy of ADT combined with RT versus ADT alone but were limited by a small number of studies and lacked comprehensive subgroup analyses to explore variations in treatment effects across different patient populations and disease characteristics. Additionally, since the publication of these reviews, several new trials have emerged, providing updated data that necessitate a re-evaluation of the existing evidence. Our meta-analysis incorporates these recent studies, allowing for a more robust and nuanced assessment of the impact of ADT plus RT on key clinical outcomes. By performing detailed subgroup analyses, we aimed to clarify which patient populations derive the greatest benefit from combined therapy, thereby addressing existing gaps in the literature and informing personalized treatment strategies for prostate cancer. Wang *et al*. conducted an indirect comparison of the efficacy of various systemic and local treatment combinations for metastatic hormone-sensitive prostate cancer [14]. Their meta-analysis included 10 randomized controlled trials, but only two compared ADT combined with RT to ADT monotherapy. The review found no significant difference in overall survival and progression-free survival between the two groups. However, overall survival was significantly improved in patients with low-volume disease, as in our analysis, in which those with LAPC and cN+ had the greatest benefit [14]. Another review by Lei *et al*. [15] documented long-term survival outcomes for ADT alone versus ADT combined with RT in locally advanced disease. A total of three RCTs (*n* = 2,344) contributed to this analysis and found that adding RT to long-term ADT improved overall survival, similar to our findings [15].

The enhanced effectiveness of combined therapy might be attributed to some underlying mechanisms. First, RT targets the tumor directly, providing localized control that may complement the systemic action of ADT, which aims to reduce circulating testosterone levels and limit tumor growth [26,27]. The combination of these modalities may yield a more comprehensive approach to tumour eradication, thereby improving patient outcomes. Moreover, in patients with LAPC or cN+ disease, the tumour is more likely to be responsive to both treatments. The localized control achieved through RT may reduce tumour burden, thereby enhancing the efficacy of ADT by mitigating the potential for cancer cells to adapt and resist hormonal therapy. This synergy might explain the observed survival benefits, particularly in these subgroups. One key aspect is the effect of ADT on the tumour microenvironment. By lowering testosterone levels, ADT may alter the tumour microenvironment, increasing the susceptibility of cancer cells to radiation [28,29]. Studies have shown that androgen deprivation can lead to increased tumour cell apoptosis and a more favorable response to RT [30,31]. Additionally, the alteration of the cell cycle dynamics induced by ADT may further sensitize prostate cancer cells to radiation [32,33].

Despite the observed survival benefits, our analysis highlights a concerning increase in the risk of complications associated with the combination of ADT and RT. Specifically, patients receiving both therapies faced a higher incidence of genitourinary and gastrointestinal complications, along with sexual dysfunction. These complications may significantly impact patients’ quality of life and overall treatment satisfaction. The increased risk of complications necessitates a nuanced approach to therapy selection, where the potential survival benefits must be weighed against the likelihood of adverse effects. Clinicians should engage in shared decision-making with patients, discussing the risks and benefits of combined therapy and considering patients’ preferences when determining the optimal treatment strategy.

This meta-analysis has several limitations that warrant consideration. First, while the pooled analysis mainly included RCTs, it also included two cohort studies, raising questions about combining different study designs. However, these cohort studies used propensity score matching to ensure similar baseline characteristics between study groups, justifying their inclusion in the pooled analysis. Further, sensitivity analysis showed that the main findings are robust when limited to randomized data. The combined RCT + cohort analyses are retained in accordance with the registered protocol, with a clear distinction between primary pooled estimates (all studies) and sensitivity estimates (RCT-only). Second, considerable heterogeneity was observed across studies for several reasons: (1) differences in study design (RCT vs propensity-matched cohort), (2) differences in disease stage (LAPC and cN vs metastatic disease), (3) variability in PFS definitions, (4) differences in radiation dose and fractionation schedules, and (5) variation in the timing of RT initiation relative to ADT. These divergent definitions likely contribute substantially to the very high I^2^ for PFS, and the PFS estimate should therefore be interpreted more cautiously than OS and PSM.

Although a random-effects model was applied to address this variability, interpreting the pooled results remains cautious. Third, while a broader range of complications would have added value to the analysis, inconsistent reporting across studies limited our ability to harmonize and pool data on complications beyond genitourinary, gastrointestinal, and sexual issues. Additionally, we could not perform a subgroup analysis based on whether ADT and RT were initiated concurrently or with ADT as neoadjuvant therapy, as in all included studies, RT began 2–3 months after ADT initiation. Lastly, our findings apply to patients aged 18–75 years and may not be generalizable to those aged 75+.

### Implications for clinical practice and future research directions

The findings have important implications for clinical practice and future research directions. First of all, we emphasize that our findings are most applicable to locally advanced high-risk non-metastatic and cN+ disease and do not support routine addition of RT to ADT in unselected metastatic patients. The findings indicate that the increased risk of complications, supported by low-certainty evidence, necessitates shared decision-making and careful counselling regarding potential toxicities. Monitoring patients for these complications is essential, with proactive management strategies implemented to address any adverse effects on quality of life. Personalizing treatment strategies based on individual patient characteristics, such as Gleason scores and baseline PSA levels, may optimize outcomes while minimizing risks. Lower Gleason score and baseline PSA <20 ng/mL are associated with better survival and PFS outcomes with ADT plus RT, suggesting these factors may help identify patients who derive greater benefit. Similarly, contemporary reviews in the post-prostatectomy, node-positive setting report that adjuvant RT, with or without ADT, improves oncological outcomes in carefully selected patients with nodal metastases, supporting the broader principle that locoregional RT adds value in biologically high-risk disease [34].

There is a need for longitudinal studies to identify biomarkers that may predict favourable responses to combined therapy. Such information could lead to more effective patient stratification and personalized treatment plans. Additionally, mechanistic studies exploring the biological interactions between ADT and RT will deepen our understanding of treatment synergies. Comparative effectiveness research should evaluate the efficacy of ADT and RT relative to emerging modalities, while patient-centered outcomes research should focus on quality of life, mental health, and treatment satisfaction.

The findings are consistent with and reinforce the current guidelines that recommend ADT plus radiotherapy to be retained for high-risk non-metastatic and clinically node-positive prostate cancer. It is briefly noted that, in the era of androgen receptor pathway inhibitors (e.g., abiraterone, enzalutamide, apalutamide) and intensified systemic regimens, the key question is increasingly how best to integrate local radiotherapy with systemic therapy rather than whether RT should be added to ADT at all. Recent narrative reviews and guideline-focused articles underscore this shift, highlighting ongoing trials of RT combined with ADT, androgen receptor pathway inhibitors, or chemotherapy in very high-risk and metastatic hormone-sensitive settings [35]. Future research should clarify the role of RT when systemic therapy is intensified and should investigate combined strategies in well-defined risk groups.

## Conclusion

Our analysis demonstrated that combining ADT with radiation therapy significantly improved overall survival, progression-free survival, and reduced prostate-specific mortality compared to ADT alone in patients with prostate cancer, particularly those with locally advanced or clinically node-positive disease. However, this combination also led to a higher incidence of genitourinary, gastrointestinal, and sexual complications. While these findings suggest a strong therapeutic benefit for selected patients, they come with a trade-off in terms of increased adverse effects. Given the limitations of study heterogeneity and moderate certainty of evidence, because (1) the number of contributing studies was small, (2) reporting of toxicity, genitourinary, gastrointestinal, and sexual complications was non-standardized, and 3) confidence intervals were wide, further research is essential to refine treatment protocols, explore optimal timing of ADT and RT, and better define patient subgroups who might benefit most from this combined approach. It is emphasized that the findings are most applicable to high-risk non-metastatic and cN+ disease and do not support routine addition of RT to ADT in unselected metastatic patients.
