# Supplementary material for: Efficacy of androgen deprivation therapy (ADT) in combination with radiation therapy, compared to ADT alone in patients with high-risk prostate cancer: an updated systematic review and meta-analysis
Source: J Med Life. 2026 Feb;19(2):98–107. doi: 10.25122/jml-2025-0140 (PMC13059460; doi:10.25122/jml-2025-0140)
Supplement: Supplementary file 1 [file JMedLife-19-098-s001.pdf]

Supplementary Table 1. Search strategies employed in identifying studies for the review

Tentative search strategy for PubMed

("prostate cancer"[Title/Abstract] OR "prostatic neoplasms"[Title/Abstract]) AND ("Androgen Antagonists"[MeSH Terms] OR "Androgen Deprivation Therapy"[Title/Abstract] OR "GnRH agonist"[Title/Abstract]) AND ("Radiotherapy"[Title/Abstract] OR "external beam radiotherapy"[Title/Abstract] OR "IMRT"[Title/Abstract] OR "SBRT"[Title/Abstract]) AND ("treatment outcome"[MeSH Terms] OR "mortality"[MeSH Terms] OR "survival"[MeSH Terms] OR "progression-free survival"[Title/Abstract])

Tentative search strategy for Scopus

(TITLE-ABS-KEY("prostate cancer" OR "prostate neoplasms" OR "prostatic carcinoma")) AND (TITLE-ABS-KEY("androgen deprivation therapy" OR "androgen suppression" OR "GnRH agonist" OR "ADT")) AND (TITLE-ABS-KEY("radiotherapy" OR "radiation therapy" OR "external beam radiotherapy" OR "IMRT" OR "SBRT" OR "brachytherapy")) AND (TITLE-ABS-KEY("clinically node positive" OR "N1 prostate cancer" OR "lymphatic metastasis")) AND (TITLE-ABS-KEY("monotherapy" OR "ADT alone")) AND (TITLE-ABS-KEY("treatment outcome" OR "mortality" OR "survival" OR "progression-free survival" OR "overall survival"))

Tentative search strategy for Web of Science

TS=("prostate cancer" OR "prostate neoplasms" OR "prostatic carcinoma") AND TS=("androgen deprivation therapy" OR "androgen suppression" OR "GnRH agonist" OR "ADT") AND TS=("radiotherapy" OR "radiation therapy" OR "external beam radiotherapy" OR "IMRT" OR "SBRT" OR "brachytherapy")AND TS=("clinically node-positive" OR "N1 prostate cancer" OR "lymphatic metastasis")AND TS=("monotherapy" OR "ADT alone") AND TS=("treatment outcome" OR "mortality" OR "survival" OR "progression-free survival" OR "overall survival")
Tentative search strategy for Embase
('prostate cancer'/exp OR 'prostate neoplasms' OR 'prostatic carcinoma')AND ('androgen deprivation therapy'/exp OR 'androgen suppression' OR 'GnRH agonist' OR 'LHRH agonist' OR 'ADT') AND ('radiotherapy'/exp OR 'radiation therapy' OR 'external beam radiotherapy' OR 'IMRT' OR 'SBRT' OR 'brachytherapy') AND ('node positive' OR 'clinically node positive' OR 'N1 prostate cancer' OR 'lymph node metastasis') AND ('monotherapy' OR 'ADT alone') AND ('treatment outcome'/exp OR 'mortality'/exp OR 'survival'/exp OR 'progression-free survival' OR 'overall survival')

Supplementary Table 2. Author's judgements about study quality using the Newcastle Ottawa Risk of Bias Assessment tool

	Lin CC *et al.* (2015)	Bekelman *et al.* (2015)
**Representativeness/appropriateness of participant selection**<br>Random or consecutive recruitment=Y<br>Convenience sample=N<br>Not reported or unclear	Y	Y
**Control for baseline differences in cohorts**<br>Similarity of groups at baseline or adjustment in analyses=Y<br>No attempt to control or adjust=N<br>Not reported=NR	Y	Y
**Loss to follow-up**<br>Explanation provided for loss of participants and/or intention to treat=Y<br>No explanation =N	Y	Y
**Masking of exposure to outcomes assessor**<br>Description of masking=Y<br>No masking or no description =N	N	N
**Ascertainment of condition**<br>Description of ascertainment/diagnostic criteria=Y<br>No description or patient self-report=N	Y	Y
**Documentation of other treatment modalities**<br>Documentation=Y<br>No documentation=N	N	N
**Extent to which valid outcomes are described**<br>Adequate description of outcome=Y<br>Insufficient detail regarding outcome or follow-up time=N	Y	Y

© 2026 by the authors. This article is an open access article distributed under the terms and conditions of the Creative Commons Attribution (CC BY 4.0) license.

Supplementary Table 2. Continued. Author's judgements about study quality using the Newcastle Ottawa Risk of Bias Assessment tool

	Lin CC *et al.* (2015)	Bekelman *et al.* (2015)
**Prespecification of harms, mode of harms collection**<br>Description of a list of harms assessed or monitoring=Y<br>No such description or passive harms collection=N<br>No adverse events reported=NA	Y	Y
**Financial Conflict of interest (COI)**<br>Funding source reported=Y<br>Funding source not reported=N	Y	Y
**Total score**	7	7

Supplementary Table 3. Quality of the pooled evidence using the GRADE assessment

	Number of studies with design	Certainty of the evidence (GRADE)	Effect size (95% CI); I^2
Overall survival	*n* = 7<br>(5 RCT; 2 cohort)	⊕⊕⊕◯<br>Moderate [a]	HR 0.75,<br>(0.63 to 0.90); 86.5%
Prostate specific mortality	*n* = 5<br>(4 RCT; 1 cohort)	⊕⊕◯◯<br>Moderate [a]	HR 0.52,<br>(0.34 to 0.78); 93.2%
Progression free survival	*n* = 5<br>(All RCTs)	⊕⊕⊕◯<br>Moderate [a]	HR 0.41,<br>(0.20 to 0.84); 98.2%
Genito-urinary complications	*n* = 3<br>(All RCT)	⊕⊕⊕◯<br>Low [b]	RR 1.80,<br>(1.15 to 2.82); 68.8%
Gastro-intestinal complications	*n* = 4<br>(All RCT)	⊕⊕⊕◯<br>Low [c]	RR 4.18,<br>(1.46 to 11.9); 83.7%
Sexual dysfunction	*n* = 2<br>(All RCT)	⊕⊕⊕◯<br>Low [d]	RR 1.10,<br>(1.02 to 1.18); 0.0%

a Downgraded one-level for serious inconsistency (high heterogeneity)
b Downgraded two-levels for serious inconsistency (high heterogeneity) and sample size less than optimal information size (OIS) (serious imprecision)
c Downgraded two-levels for serious inconsistency (high heterogeneity) and uncertainty due to wide-confidence intervals (imprecision)
d Downgraded two-levels for serious inconsistency (small number of studies); serious imprecision (suboptimal sample size)

RR, relative risk; HR, hazard ratio; RCT, randomized controlled trial

© 2026 by the authors. This article is an open access article distributed under the terms and conditions of the Creative Commons Attribution (CC BY 4.0) license.

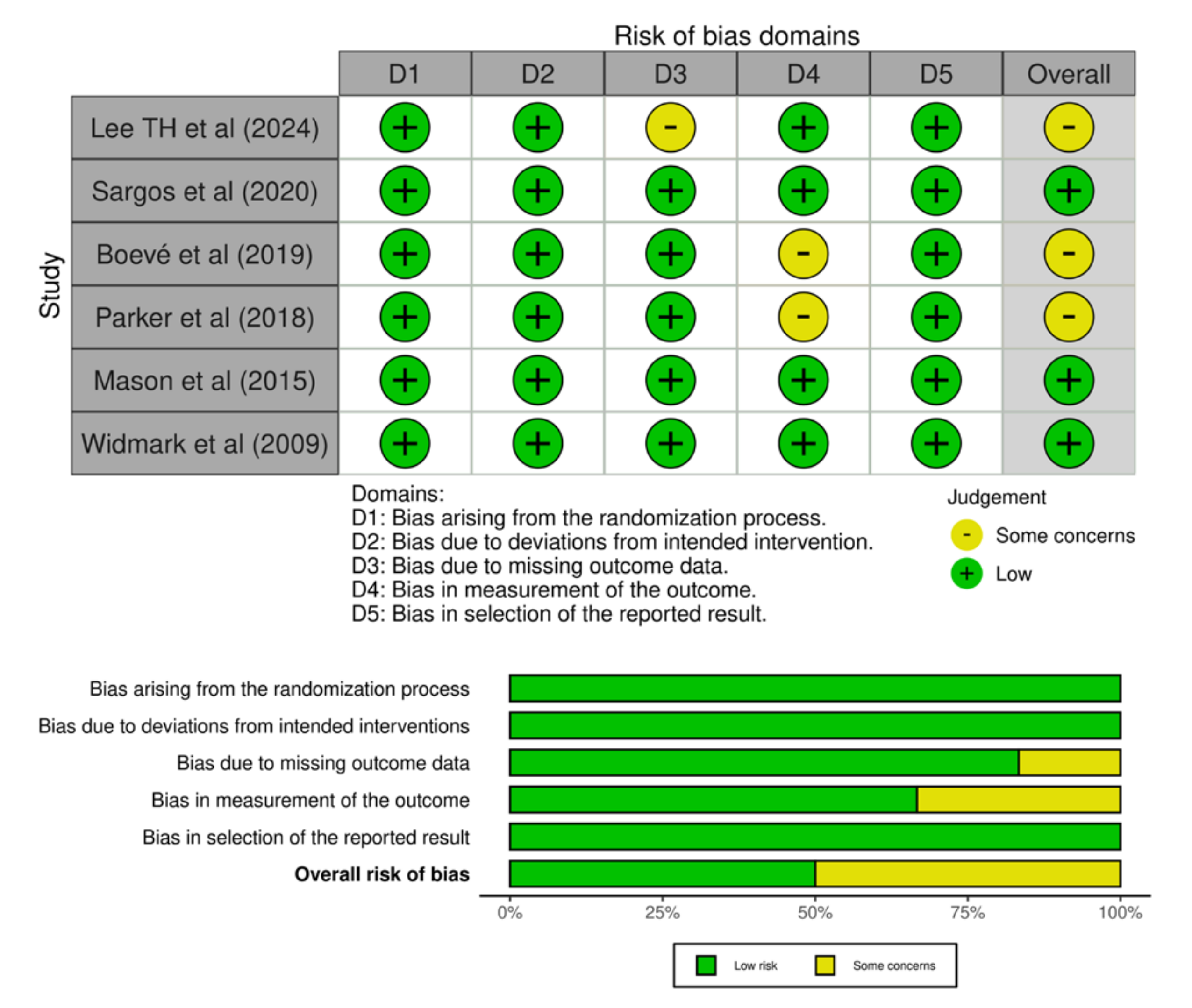

Supplementary Figure 1. Judgement of the risk of bias among the included randomized controlled trials

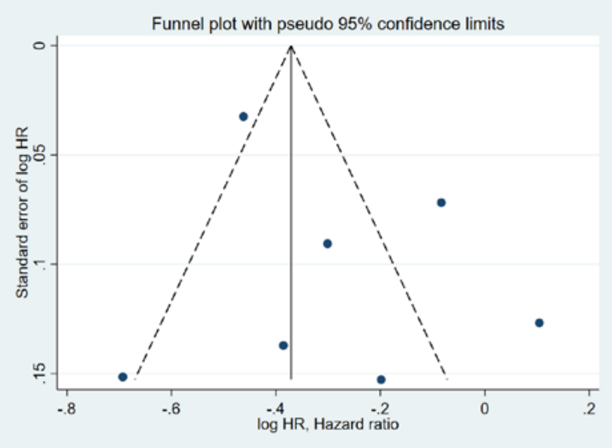

Supplementary Figure 2. Funnel plot for publication bias comparing overall survival in those receiving ADT along with RT, compared to those receiving only ADT

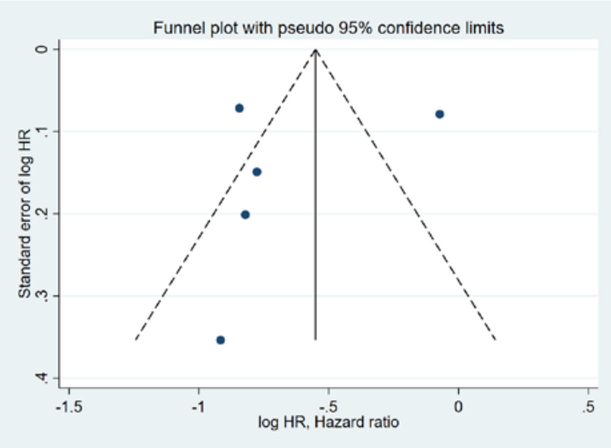

Supplementary Figure 3. Funnel plot for publication bias comparing prostate specific mortality in those receiving ADT along with RT, compared to those receiving only ADT

© 2026 by the authors. This article is an open access article distributed under the terms and conditions of the Creative Commons Attribution (CC BY 4.0) license.

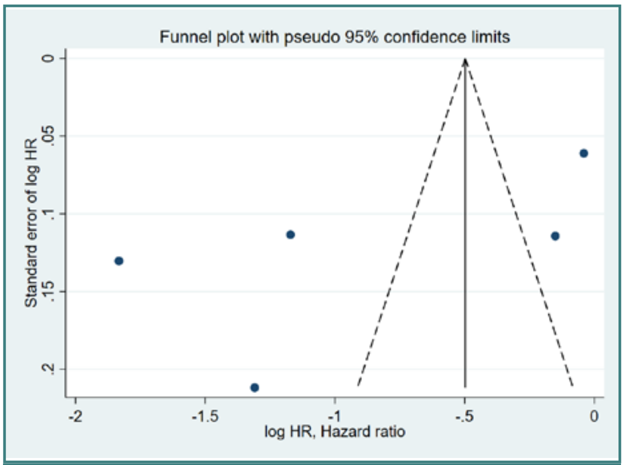

Supplementary Figure 4. Funnel plot for publication bias comparing progression free survival in those receiving ADT along with RT, compared to those receiving only ADT

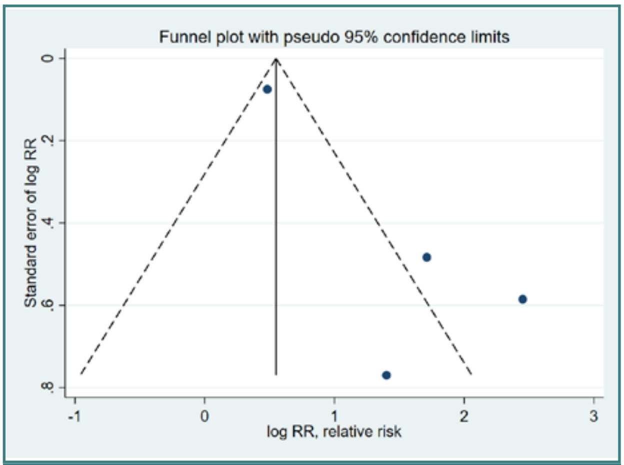

Supplementary Figure 6. Funnel plot for publication bias comparing risk of gastrointestinal complications in those receiving ADT along with RT, compared to those receiving only ADT

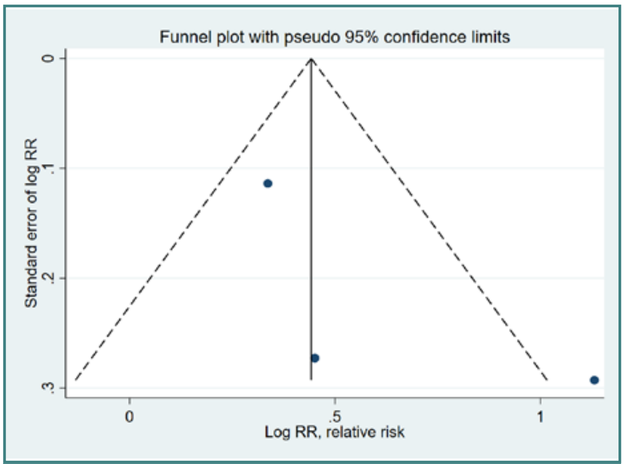

Supplementary Figure 5. Funnel plot for publication bias comparing risk of genitourinary complications in those receiving ADT along with RT, compared to those receiving only ADT

© 2026 by the authors. This article is an open access article distributed under the terms and conditions of the Creative Commons Attribution (CC BY 4.0) license.
